# Editorial: Plant Genome Editing – Policies and Governance

**DOI:** 10.3389/fpls.2020.00284

**Published:** 2020-03-11

**Authors:** Joachim Schiemann, Jürgen Robienski, Stephan Schleissing, Armin Spök, Thorben Sprink, Ralf Alexander Wilhelm

**Affiliations:** ^1^Julius Kühn-Institute (JKI), Institute for Biosafety in Plant Biotechnology, Quedlinburg, Germany; ^2^Centre for Ethics and Law in the Life Sciences, Leibniz University Hannover, Hanover, Germany; ^3^Ludwig-Maximilians-Universität München, Evangelisch-Theologische Fakultät, Munich, Germany; ^4^Science, Technology and Society Unit, Graz University of Technology, Graz, Austria

**Keywords:** CRISPR/Cas9, genome edited plants, biosafety, agriculture, policy and legislation

Genome editing and modification techniques are tools for sequence-specific changes in the plant genome. These techniques enable breeders to introduce single point mutations or new DNA sequences at a specific location in the plant genome thus for the first time enabling the precise modulation of traits of interest with unprecedented control and efficiency. The advent of genome editing has evoked enthusiasm but also controversy, creating regulatory and governance challenges worldwide. In this scenario, the Research Topic “Plant Genome Editing—Policies and Governance” aimed at collecting articles on the latest advancements and future targets of genome editing, as well as contributions addressing the regulatory, social and socioeconomic aspects, the ethics, risk assessment, management, and biosafety researches. In the following, key ideas contributed to this Research Topic are summarized which serve to illustrate the broad and complex landscape of ideas that must be addressed for plant genome editing to succeed.

## The Context—Genome Editing in Agriculture

The review article by Sedeek et al. provides a broad perspective on how plant genome editing can improve crop traits in a targeted manner. The paper highlights the TALEN and CRISPR/Cas approaches providing a general overview on the historical development of the techniques and the problems which have been addressed by targeted genome editing. It focuses on practical examples improving abiotic and biotic stress resistance as well as the improvement of yield and nutritional values. Furthermore, a short excurse provides a short overview on the regulation of genome edited crops in the US and Europe.

The paper by Nadakuduti et al. also deals with targeted improvement of crops with emphasis on improving clonally propagated crops—esp. polyploids—with a special focus on potato. It provides a general overview about the delivery of genome editing tools into plants and stresses special challenges associated with genome editing in clonally propagated crops with potato as a practical example. The authors further provide a list of clonally propagated crops which have been improved by genome editing and traits which have been addressed in the individual crops.

Metje-Sprink et al. present a special application of genome editing in crops in which no DNA is used for targeted genome modification. The authors present the different methods of performing DNA-free genome editing and current applications of DNA-free genome editing in the plant sector by providing a list of DNA-free genome applications based on a systematic literature search. Furthermore, an overview about the current and potential future delivery methods of DNA-free genome editing reagents is provided and a comprehensive overview on the current regulation of genome editing in a global perspective is given.

## Genome Editing Policy in Europe

On 25 July 2018, the European Court of Justice ruled on the interpretation of the definition of the term “genetically modified organism” in the GMO Directive 2001/18/EC. It follows from the ruling that all organisms produced by genome editing are subject to the legal framework applicable to release, placing on the market, labeling, and traceability of GMOs. In their recently published statement “Toward a scientifically justified, differentiated regulation of genome edited plants in the EU” (https://www.leopoldina.org/uploads/tx_leopublication/2019_Stellungnahme_Genomeditierte_Pflanzen_web_02.pdf), German science academies and the German Research Foundation conclude that, “due to the mounting divergence between scientific progress and legal standardization, the primarily process-based European regulatory approach is no longer justifiable” and that “potential risks can only emanate from the modified traits of the organism as a product of the breeding process, and not from the process itself.” Consequently, the statement proposes—as a first step—to amend the European genetic engineering regulation in the short term. “In a second, long term step, the legal framework should be fundamentally overhauled to place the focus on novel traits and features of an organism that are relevant to the environment, health, and nature conservation, not on the underlying breeding process.”

Legal and procedural uncertainties regarding genome edited organisms and possible ways forward for European GMO policy are described by Wasmer. He proposes that in a first step “the authorization procedure for GMO release can be tailored to different types of organisms by making use of existing flexibilities in GMO law.” Since European competitiveness and research in green biotechnology will suffer if the problems of current GMO law are ignored, in a second step “any way forward has to aim at amending, supplementing or replacing the European GMO Directive.”

How the genome editing policy in Europe is obstructing the development of new traits and is negatively influencing governance decisions and trade worldwide is described by Jouanin et al. for wheat with hypoimmunogenic gluten and by Fritsche et al. for New Zealand. Wheat with hypoimmunogenic gluten exemplifies the potential of genome editing for improving crops for human consumption where conventional breeding cannot succeed. Due to strict regulation of unintended risks at the expense of reducing the existing immunogenicity risks of patients these healthy products may become available in other parts of the world but not in Europe. Jouanin et al. strongly recommend implementing the innovation principle and argue that “Responsible Research and Innovation, involving stakeholders including patient societies in the development of gene-editing products, will enable progress toward healthy products and encourage public acceptance.” After discussing the potentials and the current regulation of genome editing in New Zealand, Fritsche et al. emphasize that for the global competitiveness of a predominantly food exporting country like New Zealand it is important that innovative technologies such as genome editing are supported by modern legislation.

With his opinion on the “politicization of the precautionary principle,” Aerni has put his “finger in the wound” of the debate on genetic engineering in Europe, which is characterized more by fear than expertise. At the same time, he discusses which consequences it can have for Europe, also in view to world trade, when the precautionary principle in genetic engineering legislations is abused as an argument for avoidance and an instrument of prevention without a science-based risk assessment.

The controversial debate whether at all and how to regulate genome edited plants has essentially led to the formation of two opposing schools of thoughts. Those who consider (certain types of) genome edited plants of low or negligible risks and argue for no or less regulation and those who highlight uncertainties and knowledge gaps and ask for same or similar regulations as for GMOs. The contributions by, Eckerstorfer et al. and Agapito-Tenfen et al. follow the latter type of thoughts. Against the backdrop of calls for regulatory reform in the EU Eckerstorfer et al. argue in favor of establishing a case-specific risk assessment for genome edited plants within the existing regulatory and biosafety framework. They suggest the EFSA guidance documents on GMO risk assessment to be updated allowing the risk assessment to be tailored to the level of uncertainties to be expected—depending on the novelty of trait / plant-use combinations, depth of genetic intervention, etc. This might also allow for a “risk assessment light” in case of minimal changes and of familiarity with a given trait/plant-use. A similar view is held by Agapito-Tenfen et al.. They conclude that a broader societal consensus is necessary for proceeding with genome editing and that research and innovation need to be governed not only by biosafety but also by societal needs, ethical principles, and sustainable development.

By comparing existing regulatory frameworks in the EU and non-EU countries, Eckerstorfer Engelhard et al. conclude that genome edited plants pose challenges for both process-triggered regulations (such as in the EU) and product-triggered systems (such as in the USA) and that eventually judicial and/or political decisions are needed to clarify if genome edited plants are covered by existing regulations. These still ongoing decision-making processes, however, are heading in very different directions, resulting in complex geographical patterns of different regulations. As harmonization is likely to take time and in order not to hamper international trade, they suggest an international public register for all GMOs including also all nGM in all jurisdictions—whether they are regulated or not.

The analysis of Bartkowski and Baum focusses on two main types of public action to express dissatisfaction, purchasing decisions as consumers (exit) and expressing views in deliberative settings (voice). According to their analysis the criticism on genome edited plants could represent a delayed response on the part of consumer-citizens to previous grievances, specifically because of their previously limited options to express their views. Following their line of thoughts, calls from both science and industry to reduce options for exit (by arguing that labeling is not possible or not necessary) might increase the level of citizen-consumer dissatisfaction. The authors suggest to extend the options for deliberation when further developing the regulatory framework with respect to genome edited plants. At the same time, they acknowledge the limitations and weaknesses of such practices, such as the constraints of power dynamics and the role of emotions. Further progress in application of the exit–voice framework can prove useful by, inter alia, helping to establish the preconditions and institutional forms necessary for such strategies to be able to effectively express (and resolve) the sources of popular dissatisfaction with the food sector.

## Alternative Governance Approaches

The disruptive energy of genome editing in plant biotechnology initiated discussions about the appropriateness of legal frameworks in many countries. Wolt and Wolf provide a generic overview of the US Coordinated Framework for Biotechnology and implications for further decision making. Though in the USA products derived from biotechnology are widely not considered “risky” because of the technology, societal uncertainties about applications of genome editing led regulators “to seek ways whereby these uncertainties may be addressed through redefinition of those products of biotechnology that may be subject to regulatory assessments.”

Societal uncertainty arises with regards to biosafety and biosecurity as reported by Fears and ter Meulen from a workshop in Hanover, Germany, in 2017. The workshop discussed potential benefits and biosecurity concerns associated with genome editing with regards to applications in human cells, agriculture, gene drives, and microbiology. The authors highlight that “it is crucial for the scientific community to share and implement good practice in self-regulation.” Sharing perspectives, facilitating information exchange, and identifying priorities for further research in biosafety and biosecurity are suggested for the scientific and biosecurity communities.

Hudson et al. discuss that modern technologies such as genome editing are not necessarily incompatible with cultural concepts that include living in harmony with nature and a special sense of responsibility for the conservation of nature. Using the example of the Maori in New Zealand, they convey an indigenous perspective and the importance of including indigenous values in the acceptance of new technologies such as genome editing in this population group.

Regulatory uncertainty around new breeding techniques is described by Lassoued et al. The success of these techniques “is not guaranteed at the scientific level alone: political influences and social acceptance significantly contribute to how crops will perform in the market.” Using survey data, Lassoued et al. report results from an international panel of experts regarding the institutional and social barriers that might impede the development of new technologies. “Survey results clearly indicate that regulatory issues, social, and environmental concerns are critical to the success of precision breeding.”

## Detection/Enforcement

Genetic modifications that occur with some likelihood through natural processes or conventional breeding efforts can hardly be distinguished from equal modifications derived by genome editing. As explained by Grohmann et al. there are several methods and approaches available to detect small differences between gene sequences (e.g., to a reference genome). But a mere sequence difference tells little about the underlying process or techniques. Extended (typical) detailed sequence information from genome edited reference organisms would be necessary to identify an underlying technical intervention with sufficient certainty. The actual accessible information, technical detection limits, natural variation in the field, and costs make it practically impossible to track and identify unwanted traces of genome edited plants in traded commodities.

## Triggers to Guide Appropriate and Proportionate Governance

In many jurisdictions the extent to which genome edited organisms fall under specific regulatory provisions depends on the genetic characteristics of the edited organism, and whether the changes introduced in its genome do (or do not) occur naturally. Custers et al. provide a number of key considerations to assist with this evaluation as well as a guide of concrete examples of genetic alterations with an assessment of their natural occurrence. “These examples support the conclusion that for many of the common types of alterations introduced by means of genome editing, the resulting organisms would not be subject to specific biosafety regulatory provisions whenever novelty of the genetic combination is a crucial determinant.”

## Social and Socioeconomic Aspects

In their research paper “New Plant Breeding Techniques [NPBT] Under Food Security Pressure and Lobbying” Shao et al. show that more strict regulations on the approval and use of NPBT will have negative implications for food security and that the costs of food production increase, decreasing the overall supply of food. While decision makers are exposed to lobbying and lobby groups can influence the regulation, it is important to recognize that lobbying is not only done by one group. “The more policy makers consider implications for food security, the less they will be influenced by lobby groups. In the case of NPBTs, the implication is that supporters of the technology have to lobby less than opponents or if they lobby, they will stress the importance of NPBTs for food security.”

## Ethics

Ethical deliberations on the regulation of genome editing reflect the social und normative conditions for the acceptance of molecular breeding technologies. This involves both the justification of normative principles and the analysis of life-world perceptions and different interests that play a role in the implementation of plant genome editing. The first aspect is dealt with in the article by Rippe and Willemsen. In response to the objection that the idea of precaution cannot be rationally justified in the end, the authors argue “for the ethical obligation to apply precautionary measures,” provided that there is a plausible scientific justification for the fear of serious damage to health and the environment. In contrast to this position, three other contributions emphasize the limits of a mere focus on risk issues in the question of social acceptability. Hamburger identifies the different interests of the stakeholders and discusses existing regulatory concepts “that are designed to facilitate a weighing and balancing of different interests or to achieve at least a mutual effectiveness of conflicting normative criteria.” Bogner and Torgersen are skeptical about the existing instruments of the Precautionary Principle (PP) and the concept of Responsible Research and Innovation (RRI). While the PP stimulates above all the expert discourse on risk issues, RRI focuses on a participatory dialogue on values in agriculture, in which existing conflicts of interest nevertheless cannot be overcome. Rather than leaving political decisions to technical risk assessment or ethics and public awareness, they argue for “re-establishing a broad yet sober process of opinion formation and informed decision-making in agricultural policy.” Bechtold is also critical of the narrow focus on risk issues in the discourse on genome editing. She argues for a comprehensive deliberation of values which allow for individual decisions within our value system. As an example, she refers to food labeling and consumer choice as “an institution to support communication about values and to broaden the perspective on the agricultural use of genome editing and its products.”

Since agriculture faces major challenges to deliver food and nutrition security the more sustainable production of more food requires the development of crops that will contribute significantly to attaining multiple Sustainable Development Goals. Plant genome editing could play a key role in developing these crops provided that accompanying the rapid scientific progress also policy and governance problems will be solved on national and international level. This Research Topic will contribute to shape the technology and its future use.

## Author Contributions

All authors contributed equally to the preparation of this editorial.

### Conflict of Interest

The authors declare that the research was conducted in the absence of any commercial or financial relationships that could be construed as a potential conflict of interest.

